# Supplementary material for: Identification of photoperiod-induced specific miRNAs in the adrenal glands of Sunite sheep (Ovis aries)
Source: Front Vet Sci. 2022 Jul 22;9:888207. doi: 10.3389/fvets.2022.888207 (PMC9354845; doi:10.3389/fvets.2022.888207)
Supplement: Supplementary file 1 [file Data_Sheet_1.ZIP › Supplementary file/miRDeep2 file.zip!/miRDeep2 file/Know_Structure/SP42A3/oar-mir-103.pdf]

miRBase precursor : oar-mir-103
Total read count : 16284
oar-miR-103 read count : 16282
remaining reads : 2

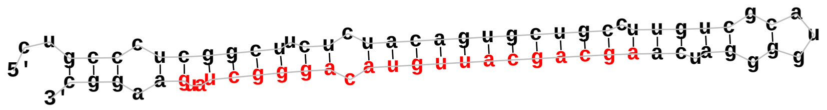

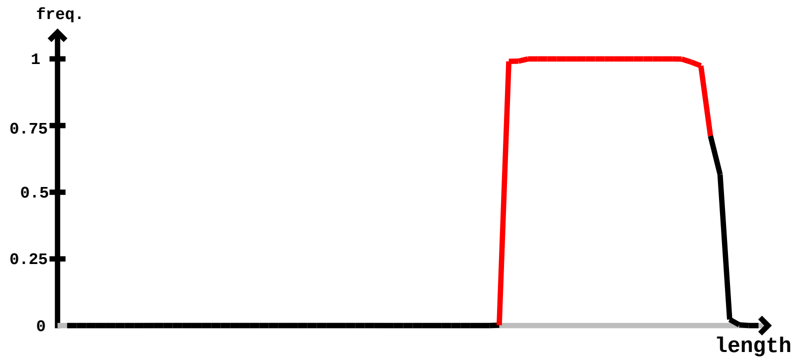

oar-miR-103

```
5'- cugcccucggcuucucuacagugcugccuugucgcauggggaucaagcagcauuguacagggcuaugaaggc   -3'    exp
    ..(((.((((((.((.(((((((((((.(((((.(...).)).))))))))))))))).))))))..)).)))   reads     mm     sample
    ........ggcuucucuacagugcugccuugu........................................   1         0      seq
    ..........cuucuUuacagugcugccuug.........................................   1         1      seq
    ............................................aagcagcauuguacagggcuau......   1         0      seq
    ............................................aagcagcauuguacagggcuaug.....   2         0      seq
    ............................................aagcagcauuguacagggcuauga....   17        0      seq
    ............................................aagcagcauuguacagggcuaugaa...   2         0      seq
    .............................................agcagcauuguacagggc.........   10        0      seq
    .............................................agcagcauuguacagggcu........   185       0      seq
    .............................................agcagcauuguacagggcua.......   224       0      seq
    .............................................agcagcauuguacagggcuau......   4273      0      seq
    .............................................agcagcauuguacagggcuaug.....   2326      0      seq
    .............................................agcagcauuguacagggcuauga....   8738      0      seq
    .............................................agcagcauuguacagggcuaugaa...   314       0      seq
    .............................................agcagcauuguacagggcuaugaaA..   37        1      seq
    .............................................agcagcauuguacagggcuaugaag..   3         0      seq
    ..............................................gcagcauuguacagggcuau......   4         0      seq
    ..............................................gcagcauuguacagggcuaug.....   2         0      seq
    ..............................................gcagcauuguacagggcuauga....   7         0      seq
    ..............................................gcagcauuguacagggcuaugaa...   1         0      seq
    ...............................................cagcauuguacagggcuau......   6         0      seq
    ...............................................cagcauuguacagggcuaug.....   19        0      seq
    ...............................................cagcauuguacagggcuauga....   97        0      seq
    ...............................................cagcauuguacagggcuaugaa...   9         0      seq
    ...............................................cagcauuguacagggcuaugaag..   1         0      seq
    ...............................................cagcauuguacagggcuaugaaA..   3         1      seq
    ................................................agcauuguacagggcuauga....   1         0      seq
```
